# Harm reduction measures employed by people using opioids with suspected fentanyl exposure in Boston, Baltimore, and Providence

**DOI:** 10.1186/s12954-019-0311-9

**Published:** 2019-06-24

**Authors:** Saba Rouhani, Ju Nyeong Park, Kenneth B. Morales, Traci C. Green, Susan G. Sherman

**Affiliations:** 1 Department of Health, Behavior, and Society, Johns Hopkins Bloomberg School of Public Health, 624 N Broadway, Baltimore, MD 21205 USA; 2 Miriam Hospital and Alpert Medical School of Brown University, Providence, RI USA

**Keywords:** Fentanyl, Overdose, Harm reduction, Drug checking

## Abstract

**Background:**

Exposure to potent synthetic opioids such as illicitly manufactured fentanyl (IMF) has fueled the escalating overdose crisis in the USA, particularly in the east coast. Drug checking services, which allow people who use drugs (PWUD) to learn about the contents of their drugs, remain limited and even criminalized in many states. Further, there is a persistent belief that PWUD are not willing or able to change their behaviors despite being aware of their potential exposure to fentanyl through drug use.

**Methods:**

We conducted a multi-site cross-sectional study among PWUD to assess what behaviors, if any, were employed in the case of suspected fentanyl exposure, and the correlates of engaging in harm reduction behaviors (HRB). PWUD (*N* = 334) were recruited in Boston (*n* = 80), Providence (*n* = 79), and in Baltimore (*n* = 175). At the time of the survey, no legal drug checking services were available in these cities.

**Results:**

The majority of PWUD (84%) expressed concern about fentanyl. Among those who suspected fentanyl exposure prior to using their drugs (*n* = 196), 39% reported employing HRB including using less of the drug (12%) or abstaining altogether (10%), using more slowly (5%), and doing a tester shot (5%). In adjusted logistic regression models, the odds (aOR) of practicing HRB after suspecting fentanyl exposure were increased among PWUD who were non-White (aOR 2.1; *p* = 0.004) and older (aOR 1.52 per decade of age; *p* < 0.001). Daily injection (aOR 0.50; *p* < 0.001), using drugs in public (aOR 0.58; *p* = 0.001), using drugs alone (aOR 0.68; *p* < 0.001), and experiencing multiple recent overdoses (aOR 0.55; *p* < 0.001) were associated with decreased odds of practicing HRB.

**Conclusions:**

These data illustrate that PWUD employ a number of practices to reduce overdose risk in a context of unknown drug purity and content. Results may also guide efforts to identify early adopters of drug checking services and engage them in peer-outreach to target the most socially and structurally vulnerable PWUD, who are not reporting behavior change, with harm reduction messaging.

## Introduction

Drug overdoses have been increasing at unprecedented rates in recent years and are now a leading cause of death and driver of reduced life expectancy in the USA [1]. The emergence and expansion of illicitly manufactured fentanyl (IMF), fentanyl analogues, and other synthetic opioids have rapidly escalated this crisis [2]. IMF and its analogs are 50–100 times stronger than heroin and are currently the most common substances involved in overdose [3], accounting for 40% of the 72,000 drug overdose deaths in 2017 alone [4]. The northeastern region of the USA has acutely felt the impact of this crisis [5]. In Maryland, the total number of drug overdoses increased for the seventh year in a row and the number of fentanyl deaths increased by 42% between 2016 and 2017 [6]. In the same year, IMF was estimated to account for 64% of all overdose deaths in Rhode Island and 90% of all fatal opioid overdoses in Massachusetts [7–9]. This crisis shows little sign of abating; the Centers for Disease Prevention and Control has reported significant increases in fentanyl-related overdose deaths in other regions of the country and in 2018 issued a health alert highlighting the growing list of illicit drugs in which these analogs are detected, including heroin, counterfeit pills, methylenedioxymethamphetamine (MDMA), cocaine, benzodiazepines, ketamine, and methamphetamine [2].

Given the unknown purity and content of illicit street drugs and the evidence that many people who use drugs (PWUD) are uncertain of their exposure to fentanyl before use [10, 11], many have called for a harm reduction approach to this crisis [12, 13]. Expanded access to naloxone has been one successful harm reduction measure [14] and targeted programs have achieved progress in empowering PWUD as well as their friends and family with tools to reverse overdose among their peers [15, 16]. However, comparatively less focus has been placed on arming them with resources for prevention. Harm reductionists continue to advocate for the implementation of measures such as drug-checking services, which have been employed for many years in countries across Europe, Australia, and Latin America and shown success in preventing overdose deaths [17–25]. Services vary between settings but generally provide access to anonymous testing of illicit, psychoactive substances to provide PWUD with knowledge of the content and purity of their drugs and enable them to make informed decisions about use. Many programs also serve to inform public health monitoring and alert systems, which help PWUD, health workers, law enforcement, and the general public remain up to date with drug market changes. In the context of the US opioid crisis, fentanyl test strips (FTS) have been proposed as a drug-checking tool which can be used by PWUD to detect the drug at the point of use [13, 26]. Despite evidence of their sensitivity, validity, and acceptability across PWUD and other stakeholders [27–29], their legality, promotion, and availability vary between states.

A critique of employing drug-checking approaches is the belief that PWUD do not care about their own health and are unable to make rational decisions altering their behavior even with the necessary information to do so. This notion has been refuted, most extensively in literature detailing behavior change and HIV risk among PWUD [30] and more recently by PWUD themselves in the era of fentanyl adulteration [31]. Nonetheless, in the US context, skepticism around PWUD’s willingness and ability to make rational decisions persists, comprising a barrier to providing access to evidence-based interventions such as drug checking services [32]. This study aims to examine whether and how, in the absence of such services, PWUD change their drug use behaviors after suspected fentanyl exposure in three northeastern US cities—Baltimore, Maryland, Providence, Rhode Island, and Boston, Massachusetts—that have been profoundly impacted by the emergence of IMF. Understanding how PWUD respond to suspected exposure and the individual and structural factors that shape the employment of HRB is important for harm reduction advocacy and the scale up of effective interventions to reduce the risk of overdose.

## Methods

### The FORECAST study

The Fentanyl Overdose Reduction Checking Analysis Study (FORECAST) is a multi-site study measuring the validity and feasibility of drug-checking tools. The comprehensive methodology for the FORECAST study are described elsewhere [27, 28, 33, 34]; in brief, participants were recruited through syringe service programs in Providence and Boston and using targeted sampling to identify and recruit from areas with a high density of drug arrests. The survey phase of the project enrolled 334 PWUD who completed a screening followed by an anonymous, 30-min computer-assisted personal interview from May 2016 to April 2017. Participants in Boston (*n* = 80) and Providence (*n* = 79) were recruited through syringe service programs (SSP) and harm reduction services. In Baltimore, Baltimore City Police Department, drug arrest data from 2016 was used to identify regions for street-based targeted sampling [35] to recruit PWUD (*n* = 175). PWUD were eligible for inclusion if they were at least 18 years of age, spoke English, and reported the use of non-medical heroin, fentanyl, cocaine, methamphetamine, or opioid pills in the past 30 days.

At the time of the study, FTS were not being widely promoted or distributed in Boston, Providence, or Baltimore. All study participants provided informed oral consent and were compensated $25 USD upon completion. The study was approved by the Johns Hopkins Bloomberg School of Public Health and the Rhode Island Hospital Institutional Review Boards.

### Measures

Sociodemographic data (e.g., age, gender, race, homelessness, and employment status), knowledge and experience of fentanyl, and history of drug use, overdose, criminal justice interaction, and use of health and harm reduction services were ascertained. Age was analyzed as a dichotomous variable (< 35, ≥ 35) and as a continuous variable (scaled, in decades) in regression analyses. In regression, race was also considered a binary term (White/non-White) due to small sample sizes of Hispanic and mixed- or other racial groups. Polydrug use was analyzed as a binary variable denoting the use of the median number of 4 or more substances in the past 6 months. The usual setting of drug use was also considered as a binary variable, with public use inclusive of public (street, park) and semi-public spaces (stairwells, abandoned buildings, vehicles, public restrooms, shooting galleries), compared with private use in a home setting.

To define and measure the primary outcome, practicing HRB, participants (*N* = 334) were first asked whether they had ever used a drug they thought had fentanyl in it (yes/no); those who had (*n* = 256) were then asked what they did in response. Fifty-seven participants suspected contamination *after* using; of the remaining 199, 196 answered questions regarding behavior change and were included in this analysis. Survey response options for behavior change were adapted from prior studies in Rhode Island [7, 36] with input from PWUD and study staff at each site (many of whom were also PWUD). Responses were as follows: *use as originally intended* (*nothing different*); *use less than originally intended*; *go slower*; *do a tester*; *sniff/toot/smoke instead of shooting* (*not inject*); *use with someone else around*; *share/take turns when I use*; *use the drug somewhere safe* (*like where someone could find me*); *throw them out*; *give them away*; *sell them*; *get naloxone/Narcan*; *tell the dealer/supplier*; *get money back from dealer/supplier*; *beat up dealer/supplier*; *tell other people who use the same dealer/supplier*; *stop going to that dealer/supplier*; *other* (*specify*)*.* Categories were collapsed and a dummy variable was created classifying those responding “*use as originally intended*” as “no behavior change” and those reporting a change to their method of use or acquisition to minimize exposure risk (*use less; go slower; do a tester; not inject; use with others; share/take turns; use in a safer place; get naloxone/Narcan)* as practicing HRB. We additionally included behaviors that amounted to total, primary prevention (*throw away; give away; sell; get refund)* as HRB in this setting, given that they were adopted specifically in response to suspected adulteration. Responses among participants who selected “*other*” were reviewed and coded as HRB (e.g., “warn my friends,” “give stash to hospital,” “keep Narcan close by”), no behavior change (e.g., “used it anyway and felt sick,” “enjoyed it”), or excluded (responses indicating lack of knowledge before use, e.g., “overdosed”).

### Statistical analysis

We compared the frequency of reported HRB by city and across sociodemographic, drug use history, criminal justice, and health-related correlates of interest using Pearson’s *χ*^2^ tests. Trends in HRB (*p* < 0.1) in the sample were reported and correlates were modeled using multivariable logistic regression with clustered variance estimation to adjust for differences by site. We did not analyze the likelihood of behavior change according to SSP attendance in subsequent regression models due to potential bias, as the recruitment of participants occurred through SSP in two of our sites (Boston, Providence). If the distribution of HRB differed by any other correlate at a level of *p* < 0.1 using either *χ*^2^ or bivariate regression (with clustered variances), they were considered for inclusion in the final adjusted model; only the subset of items informed by existing literature and with demonstrated statistical association with HRB were included to achieve the greatest parsimony given a small sample size. We excluded “using drugs alone” from regression models due to confounding with one of the behaviors defining the outcome (“use with others”). We conducted a sensitivity analysis with additional adjustment for fentanyl preference (not shown); however, given the lack of significant influence on the outcome and the absence of other qualitative or substantial quantitative changes to model output, we omitted this variable from the final model. Analyses were conducted using Stata S/E 14.2 (College Station, TX).

## Results

Participants were predominantly male (59%) and White (47%), with age ranging from 20 to 65 years (mean 42 years). The majority of the population was homeless (69%) and lacked stable employment (88%). Sociodemographic characteristics of the sample are shown in Table 1. Across sites, 39% of individuals reported practicing HRB when suspecting fentanyl exposure, most commonly reporting behaviors such as using less (12%), abstaining from use (10%), using drugs more slowly (5%), and doing a tester shot (5%). Individuals aged 35 or older had a significantly higher prevalence of HRB relative to those below the age of 35 (24% vs. 45%, *p* = 0.007) as did Black, Hispanic, and other non-Whites compared to White participants (52% vs. 46% vs. 27%, respectively *p* = 0.004).

**Table 1:** Socio-demographic characteristics of FORECAST participants according to reported behavior change during last suspected fentanyl exposure

	Total	Used as intended	Practiced HRB	*p* value
	No. (col %)	No. (row %)	No. (row %)	
Total	196 (100)	119 (60.7)	77 (39.3)	
Study center				
Baltimore	103 (52.6)	62 (60.2)	41 (39.8)	
Boston	53 (27)	35 (66.0)	18 (34.0)	
Providence	40 (20.4)	22 (55.0)	18 (45.0)	0.552
Age				
< 35	54 (27.6)	41 (75.9)	13 (24.1)	
> 35	142 (72.4)	78 (54.9)	64 (45.1)	*0.007*
Gender				
Male	116 (59.2)	75 (64.7)	41 (35.3)	
Female	80 (40.8)	44 (55.0)	36 (45.0)	0.174
Race				
NH White	92 (46.9)	67 (72.8)	25 (27.2)	
NH Black	63 (32.1)	30 (47.6)	33 (52.4)	
Hispanic/other	41 (20.9)	22 (53.7)	19 (46.3)	*0.004*
Homeless	135 (68.9)	83 (61.5)	52 (38.5)	0.744
Legally unemployed	172 (87.8)	105 (61.0)	67 (39.0)	0.799
Drug use, past 6 months				
Heroin	182 (92.9)	109 (59.9)	73 (40.1)	0.394
Cocaine	170 (86.7)	102 (60.0)	68 (40.0)	0.601
Prescription opioid use (illicit/misuse)	87 (44.4)	52 (59.8)	35 (40.2)	0.809
Crack cocaine	143 (73)	81 (56.6)	62 (43.4)	0.055
Methamphetamine	29 (14.8)	21 (72.4)	8 (27.6)	0.162
Illicit benzodiazepine use/misuse	103 (52.6)	68 (66.0)	35 (34.0)	0.110
Alcohol	123 (62.8)	65 (52.8)	58 (47.2)	*0.003*
Polydrug use (> 4)	104 (53.1)	60 (57.7)	44 (42.3)	0.357
Overdose history				
Ever overdose				
No	55 (28. 2)	27 (4.1)	28 (50.9)	
Yes	140 (71.4)	92 (65.7)	48 (34.3)	*0.032*
Number of overdoses, past year				
None	102 (52.0)	58 (56.9)	44 (43.1)	
One to two	52 (26.5)	30 (57.7)	22 (42.3)	
Three or more	42 (21.4)	31 (73.8)	11 (26.2)	0.074
Ever witnessed a fatal overdose	91 (46.4)	56 (61.5)	35 (38.5)	0.826
Risk environment				
Injection drug use				
Never	43 (21.9)	18 (41.9)	25 (58.1)	
Less than daily	50 (25.5)	28 (56.0)	22 (44.0)	
≥ Daily	103 (52.6)	73 (70.9)	30 (29.1)	*0.003*
Usual drug use setting, past 30 days				
Private	50 (25.5)	33 (45.2)	40 (54.8)	
Public	103 (52.6)	83 (69.7)	36 (30.3)	*0.001*
Usually used drugs alone	87 (44.4)	55 (63.2)	32 (36.8)	0.521
Rushed buying, using or preparing drugs due to fear of police, past year	168 (85.7)	104 (61.9)	64 (38.1)	0.403
Sold drugs, past 3 months	118 (60.2)	77 (65.3)	41 (34.8)	0.109
Arrest, past year	102 (52)	64 (62.7)	38 (37.3)	0.544
Drug-related arrest, past year	64 (32.7)	38 (59.4)	26 (40.6)	0.789
Drug treatment and harm reduction				
Drug treatment (any), past 6 months	120 (61.2)	67 (55.8)	53 (44.2)	0.060
Medication-assisted treatment	81 (41.3)	43 (53.1)	38 (46.9)	0.066
Rehabilitation or detoxification	51 (26)	31 (60.8)	20 (39.2)	0.991
Counseling/mental health/support group	57 (29.1)	27 (47.4)	30 (52.6)	*0.014*
Carry naloxone	105 (53.6)	66 (62.9)	39 (37.1)	0.637

Significant *p* values (< 0.05) are shown in italics

### Drug use, overdose history, and risk environment

The most prevalent reported drugs used were heroin (93%), cocaine (87%), crack (73%), alcohol (63%), and benzodiazepines (53%), and 53% of the sample reported the use of more than four substances in the past 6 months. Employing HRB was less common among people who inject drugs (PWID), daily (29%), or at all in the past 3 months (44%), relative to those who did not inject (58%; *p* = 0.003). Significantly fewer participants who used drugs in public or semi-public spaces reported HRB (30%) compared with those using in private settings (55%; *p* = 0.001).

The majority (71%) of the sample had experienced overdose, and 48% had done so within the past year. The reported prevalence of HRB was lower among individuals who had a history of overdose than among those who had not (34% vs 51%; *p* = 0.032). A suggestive dose-response pattern was detected with reducing the prevalence of behavior change correlating with greater number of overdose events within the past year (*p* = 0.074).

### Drug treatment and harm reduction

Practicing HRB was higher among participants receiving drug treatment in the past 6 months relative to those who did not, though this was marginally non-significant (56% vs 44%; *p* = 0.060). When treatment was examined by category, we observed significantly higher reported HRB among PWUD with recent counseling, support group attendance, or mental health services, relative to those without (62% vs 39%; *p* = 0.014).

### Fentanyl concern, recognition, and preference among PWUD practicing HRB

The vast majority of PWUD expressed concern about fentanyl (81%), and this was higher among those who practiced HRB (94%) relative to those who did not (72%; *p* < 0.001; Table 2). PWUD who reported HRB also more frequently expressed the desire to know with more certainty whether their drugs were adulterated (91% vs 79%; *p* = 0.029). Participants expressing a preference for fentanyl (37%) had a significantly lower frequency of reporting HRB compared to those who did not (18% vs. 50%, *p* ≤ 0.001).

**Table 2:** Fentanyl concern, recognition, and preference among PWUD practicing HRB

	Total	Used as intended	Practiced HRB	*p* value
	No. (col %)	No. (col %)	No. (col %)	
Total	196 (100)	119 (100)	77 (100)	
Concern about fentanyl	157 (80.5)	85 (72.0)	72 (93.5)	*< 0.001*
Wished they could know about fentanyl presence with more certainty	160 (81.6)	92 (78.6)	68 (90.7)	*0.029*
Prefer fentanyl	73 (37.2)	59 (49.6)	14 (18.2)	*< 0.001*

Significant *p* values (< 0.05) are shown in italics

### Adjusted correlates of reported HRB

Adjusted correlates of reporting behavior change are shown in Table 3**.** After adjusting for gender and treatment history, the odds of practicing HRB increased significantly with each successive decade of life (aOR1.5; *p* = 0.001). Participants who were not White had twice the odds of reporting HRB during last suspected fentanyl exposure (aOR 2.0; *p* = 0.009). Drug use characteristics that were significantly associated with a decreased likelihood of practicing HRB in response to fentanyl exposure were daily injection (aOR 0.5; *p* < 0.001) and using in public (aOR 0.6; *p* = 0.001).

**Table 3:** Adjusted correlates of reported HRB among FORECAST participants (*n* = 196)

	Adjusted odds ratio	95% confidence interval	*p* value
Age, decade	1.49	1.18, 1.88	*0.001*
Gender			
Male	Ref		
Female	1.85	0.81, 4.26	0.144
Race			
Non-Hispanic White	Ref		
Black and/or Hispanic	2.0	1.20, 348	*0.009*
Risk environment			
Daily injection drug use	0.49	0.47, 0.51	*< 0.001*
Usual drug use setting, past 30 days			
Private	Ref		
Public	0.58	0.41, 0.51	*0.004*
Overdose history			
Multiple overdoses within the last year (> 2)	0.58	0.49, 0.68	*< 0.001*
Drug treatment and harm reduction			
Drug treatment, past 6 months (any)	1.89	0.96, 3.72	0.065

Significant *p* values (< 0.05) are shown in italics

## Discussion

This study characterized the adoption of a range of harm reduction behaviors among PWUD after suspecting fentanyl exposure in three cities with extremely high rates of fentanyl-related morbidity and mortality and limited exposure to drug checking services. Eighty-four percent of PWUD were concerned about fentanyl in their drugs, and 39% reported practicing HRB, such as doing a tester shot or using drugs more slowly or not at all when they perceived their drugs to be adulterated. These data illustrate that PWUD are concerned about their risk of exposure and employ harm reduction approaches in a context of unknown drug purity and content. Our findings can inform messaging around safe drug use in the context of fentanyl-adulterated markets with and without drug checking.

We observed a number of important factors associated with HRB. Non-White participants had twice the odds of practicing HRB in response to suspected fentanyl exposure. This is consistent with some prior work illustrating higher adoption of protective behaviors like using a new needle (i.e., lower needle sharing) [37, 38] among Black compared to White PWUD. This finding may also be reflective of the changing racial demographic of street-based people using illicit opioids, driven by the increase in White PWUD transitioning along a risk continuum from medical or nonmedical use of prescription opioids to heroin and IMF use [39]. These individuals may therefore lack access to existing social networks within the community of PWUD or within the Black community that help to spread knowledge and tools for harm reduction.

Younger individuals less frequently practiced harm reduction around suspected exposure. This is consistent with literature detailing changes in opioid initiation and use by birth cohort and may be explained by differences in drug content and availability [40]. For example, younger PWUD may be more recent drug initiates who acclimatize to the current market, which is highly saturated with fentanyl, and therefore, an environment where fentanyl exposure is common and more normalized. Indeed, data from this study have suggested that people who prefer fentanyl are generally younger, newer cohorts of PWUD (Morales et al; *under review*). However, it is important to note that even among the minority of individuals who prefer fentanyl in our study, some (18%) still also reported HRB, highlighting that PWUD may make decisions based on their perceived risks, risk setting, or scenario, rather than their preferences. Substance use and misuse literature highlight lack of perceived risk due to factors like impulsivity, lack of appreciation of consequence, and susceptibility to peer norms as a driver of adopting high-risk practices in young adulthood [41]. Data have also shown that young adults who underestimate overdose risk are less informed about resources for prevention and reversal and are often outside of networks reached by overdose prevention services [42]. This may in part be explained by observations that younger PWUD are often disconnected from older, more experienced cohorts of PWUD and are therefore more isolated from harm reduction resources and social networks that diffuse HRB [43].

We detected several markers of extreme vulnerability to overdose that were also associated with lower employment of HRB, including frequent injection drug use and use in public or semi-public spaces. This finding is consistent with the theory that forms of social and structural disadvantage interact to produce a risk environment that can limit the capacity of PWUD to reduce drug-related harm [44, 45]. This framework underscores the marginalization of these populations, whose risk environment may include broader issues like lack of social support, housing, and access to mental and social services [46] and who are at elevated risk of more extreme forms of substance use disorder and nonfatal and fatal overdose risk [11, 47]. We found an inverse relationship between overdose history and HRB, with individuals who had experienced multiple overdoses in the past year being almost half as likely to engage in HRB than those with one or fewer. It is possible that overdose frequency is a marker of overall vulnerability in the sample and like other such indicators is associated with a decreased likelihood of practicing responsive behavior change [48]. Alternatively, PWUD in this category may be more likely to overdose because they did not reduce harm when exposed to fentanyl. Given the cross-sectional design of this study, we cannot infer the direction of this relationship, though both interpretations underscore the strong link between overdose frequency and the lack of access to or adoption of harm reduction messaging and resources.

The proportion of PWUD practicing HRB in these three cities is nearly identical to that reported in a recent study assessing responsive behavior change after distribution of FTS in North Carolina [29]. After adjustment for potential confounders, Peiper and colleagues observed that PWUD obtaining results indicating fentanyl presence were five times more likely to enact behavior change than those who obtained negative results. These findings highlight the importance of conducting more research on how to increase the practice of HRB, beyond the provision of drug-checking tools. However, our findings alongside those of Peiper and others [49] serve to counter the notion that PWUD cannot enact rational decision-making when placing themselves closer to or farther from harm. Giving PWUD the tools to detect fentanyl with more certainty seems a practical and ethical extension of these observations; nonetheless, the narrative that PWUD are not making rational choices and would not reduce harm or abstain from use if they suspected fentanyl contamination was recently reasserted by one government official [32]. Legislation in many states across the country reflects this ideology, classifying FTS as drug paraphernalia [50–56], thus deterring the use of an important harm reduction tool that can inform promotion of HRB.

This study has important programmatic implications for health and outreach services. As drug checking programs are increasingly decriminalized in many contexts, these data can help to guide strategies to target PWUD as such programs are enacted. Individuals who are more concerned about fentanyl and already practicing harm reduction may be rapid adopters of expanded services and may be engaged in peer outreach and training efforts to increase knowledge, use FTS and other tools, and behavior change among fellow PWUD. This will enable PWUD who are already practicing HRB do so more in a more informed manner and can empower and engage them in peer-led overdose prevention programs. Given that the majority of PWUD reported are also selling drugs in the past 3 months, strategies to engage dealers in drug checking and other harm reduction practices may also be appropriate [57] in this context. Likewise, our findings highlight critical groups to target with harm reduction and behavior change messaging, as they are both at increased risk of overdose and exhibit low likelihood of safer practices. In particular, innovative and targeted methods are needed to engage younger PWUD in harm reduction. Finally, we observed a trend that individuals receiving drug treatment were more likely to report behavior change, highlighting their role as a point of contact to deliver life-saving messages and the importance of using such services to promote harm reduction, rather than just abstinence. This is particularly important in light of the increased risk of overdose among PWUD leaving drug treatment [58].

Our findings should be interpreted in light of study limitations. We rely on self-report to capture whether and how PWUD enact behavior change and as such data are subject to biases due to either poor recall or systematically inflated responses based on the desirability of responses. However, we note that the use of anonymous, computer-assisted surveys likely reduces the impact of the latter [59, 60]. The list of HRB included in this study was predetermined, but hardly exhaustive. Many other strategies or actions among PWUD to reduce risk of fentanyl are likely being innovated and should be examined in future research. The cross-sectional study design precludes us from making assumptions about causality or sequence of drug use, behavior change, and overdose events. Finally, we did not corroborate PWUD perceptions of whether the drugs they used were actually positive for fentanyl, and so results may be impacted by differing individual perceptions of contamination in the populations surveyed.

Finally, this work raises a number of important questions for future research. As previously noted in the literature, harm reducing behaviors are not simply determined by access to knowledge of contaminants, but multiple interacting and often upstream contingencies [13, 44]. Though our findings illustrate that PWUD are adopting some forms of HRB in the absence of drug checking, the ways drug contamination information is disseminated may be among factors that influence the extent to which they do or do not change behaviors. For example, learning of the presence of fentanyl via FTS may elicit or encourage much different responses than accessing information via media reports of overdose hotspots [61, 62]. As more forms of drug checking are considered and become available in the US setting, it will be important to continue measuring both qualitatively and quantitatively the ways in which PWUD translate information from different sources to behaviors that can reduce their risk. Further, as the supply becomes more saturated with fentanyl [63], understanding the implications for drug checking programs and HRB will be critical to enact effective overdose prevention.

## Conclusions

This study demonstrates that in three hotspots of the fentanyl epidemic, there is a will and a way to practice harm reduction when faced with fentanyl exposure and risk of death. These data complement the existing body of evidence refuting the notion that PWUD are indifferent to the value of their lives and unable to enact rational decision-making and provide channels for future research to assess how to increase HRB among PWUD at high risk of overdose. Providing PWUD with tools to protect themselves is an ethical imperative in the era of widespread adulteration of a range of substances and rampant overdose fatalities. Access to harm reduction education and services such as drug-checking should be broadly expanded in these settings and beyond.

## Data Availability

Data may be available upon request from the corresponding author. The data are not publicly available due to containing information that could compromise participant consent.

## Abbreviations

aOR: Adjusted odds ratio
FTS: Fentanyl test strips
HRB: Harm reduction behaviors
IMF: Illicitly manufactured fentanyl
MDMA: Methylenedioxymethamphetamine
PWID: People who inject drugs
PWUD: People who use drugs
SSP: Syringe service programs
